# Downregulation of miRNAs Accompanies Increased HERV-K (HML-2) Expression in Amyotrophic Lateral Sclerosis

**DOI:** 10.1007/s12035-026-05861-5

**Published:** 2026-04-17

**Authors:** Elena Rita Simula, Marta Garcia-Montojo, Mattia Canu, Vanna Chessa, Tommaso Ercoli, Elisa Ruiu, Paolo Solla, Avindra Nath, Leonardo Antonio Sechi

**Affiliations:** 1 Department of Biomedical Sciences, Division of Microbiology and Virology, University of Sassari, Sassari, Italy; 2 National Institute of Neurological Disorders and Stroke, National Institutes of Health, Bethesda, MD USA; 3 Twilight Bioscience, Inc. 100 Cummings Center. Suite 207-209, Beverly, MA USA; 4 ASL Sassari, SC Anestesia Territoriale Cure Palliatiave, 07100 Sassari, Italy; 5 Neurological Unit, AOU Sassari, University of Sassari, Viale S. Pietro 10, 07100 Sassari, Italy; 6 Complex Structure of Microbiology and Virology, University Hospital of Sassari, Sassari, Italy

**Keywords:** ALS, MiRNAs, HERV-K, HML-2, Neurodegeneration, Gene regulation

## Abstract

Amyotrophic lateral sclerosis (ALS) is a rapidly progressing neurodegenerative disease with limited treatments. Evidence suggests that reactivation of the HERV-K (HML-2) subgroup contributes to its pathogenesis. This study explores the role of microRNAs (miRNAs) in regulating HML-2 expression. We identified dysregulated miRNAs in ALS and, among them, those predicted to target the HML-2 transcript. The expression levels of selected miRNAs were validated in peripheral blood leukocytes of ALS individuals and healthy controls. Co-transfection experiments were then performed to determine the regulatory potential of these miRNAs on HML-2 expression. We found that the HML-2 envelope gene expression levels were elevated in peripheral blood mononuclear cells of ALS individuals compared to controls (*p* = 0.02), and they negatively correlated with the levels of previously identified miRNAs, which were downregulated in patients compared to healthy controls (miR-15a-3p, *p* = 0.04; miR-15a-5p, *p* = 0.01; miR-150-5p, *p* = 0.001; miR-182-5p, *p* = 0.012; miR-192-3p, *p* = 0.034; miR-221-3p, *p* = 0.011), except miR-181a-2-3p that was upregulated in ALS compared to controls (*p* = 0.006). Among these miRNAs, we found, by co-transfection, that miR-182-5p and miR-221-3p were capable of binding the HML-2 transcript. This interaction resulted in a significant downregulation of the expression of its genes, with a pronounced effect observed on the envelope gene. Our findings suggest a link between miRNAs and HML-2 expression. In particular, the observed increase in HML-2 levels in ALS may result from the downregulation of key miRNAs, such as miR-221, that normally help restrain HML-2 expression under physiological conditions.

**Supplementary Information:**

The online version contains supplementary material available at 10.1007/s12035-026-05861-5.

## Background

Amyotrophic Lateral Sclerosis (ALS) is a neurodegenerative disorder characterized by the progressive death of motor neurons. The principal clinical manifestations of ALS include paralysis of voluntary muscles, atrophy, and fasciculations. Typically, the disorder results in a life expectancy of three to five years after onset, and respiratory failure is the most common cause of fatality [1]. ALS is commonly classified according to the initial site of motor neuron involvement. Limb-onset ALS is characterized by early weakness in the arms or legs, whereas bulbar-onset ALS typically presents with speech or swallowing difficulties [2]. Each subtype has unique clinical features and disease progression, suggesting that different underlying mechanisms may contribute to the development and progression of the pathology [3].

TDP-43 proteinopathy in the brain is the hallmark of ALS, being present in approximately 97% of patients, but the molecular mechanisms of ALS pathogenesis are not yet fully understood. Pathogenic alterations are believed to involve interference with protein expression and degradation and defects in RNA processing [4]. These abnormalities result in progressive cell breakdown, disruption of axonal architecture and function, axonal retraction, and ultimately denervation of muscles [5].

Also, the etiology of ALS remains largely elusive, ranking the disorder within the spectrum of multifactorial diseases involving the interplay of multiple genetic, and environmental, factors [6]. Genetic predispositions in familial cases account for approximately 5–10% of incidence. On the other hand, sporadic ALS, which constitutes the majority of cases, hints at potential environmental triggers that could influence disease onset and progression [7]. Recent studies have provided evidence supporting that the reactivation of a specific protein encoded by Human Endogenous Retrovirus (*HERV*), namely *envelope* (HML-2 env), may play a role in the development of ALS [8–10].

HERVs are sequences of retroviral origin that compose approximately 8% of the human genome and exhibit high levels of expression in stem cells, predominantly silenced following cell differentiation [11]. Although they generally remain dormant, the reactivation of HERVs has been implicated in various neurodegenerative diseases, including ALS [12, 13]. HERV-K, and particularly the HML-2 subgroup, has nearly a hundred copies within the human genome and is the most recently acquired and transcriptionally active. It is involved in various pathological contexts, including neurodegeneration, cancer, and autoimmune diseases [14–18]. Notably, specific loci of HML-2 that encode for the envelope protein are expressed in the central nervous system of a subset of ALS individuals but not in unaffected controls [8]. Cellular and in vivo models of ALS show that overexpression of the envelope protein of HML-2 leads to neuronal death, particularly motor neurons, causing an ALS-like syndrome in transgenic mice. HML-2 env transgenic mice display progressive motor impairment and a shortened life-span [8]. Moreover, a relationship between HML-2 and TDP-43 proteinopathy has been demonstrated, pointing out mechanistic pathways for the neurotoxic effects of HML-2. HML-2 expression is positively correlated with mislocalized TDP-43 protein levels within the cortical brain tissue and human neuronal cells from iPSCs [19] and it is sufficient to induce cytoplasmic aggregation and phosphorylation of TDP-43 [20].

The potential role of HML-2 reactivation in the pathogenesis of ALS has prompted investigations into the mechanisms behind this reactivation but the exact processes involved remain elusive. One possible mechanism, that we investigate in this study, is related to microRNAs (miRNAs). The Human Genome Project disclosed that within the three billion base pairs of the human genome, merely about 2% are involved in protein-coding. This suggests that a substantial portion of the genome is involved in generating a diverse range of non-coding RNAs (ncRNAs), which are increasingly recognized for their regulatory roles not only in normal cellular processes but also in pathological conditions, including neurodegenerative diseases, cancers, and autoimmune disorders [21–24]. MiRNAs are evolutionarily conserved, tissue-specific small ncRNAs, typically ranging from 18 to 25 nucleotides in length. They are crucial post-transcriptional regulators of gene expression. They operate by binding to complementary sequences of target messenger RNAs, leading, most often, to suppression of protein synthesis. This suppression occurs through mechanisms such as mRNA destabilization or inhibition of mRNA translation, effectively downregulating gene expression [25]. Notably, a single miRNA can regulate multiple genes, while the expression of a particular gene can be controlled by a complex network of interacting miRNAs [26]. Numerous studies have demonstrated that the biogenetic pathways of miRNAs are disrupted in ALS. Notably, Campos-Melo et al. [27]. and Figueroa-Romero et al. [28]. have reported alterations in miRNA profiles. Both familial ALS (fALS) and sporadic ALS (sALS) individuals exhibit decreased levels of specific miRNA subsets when compared to healthy controls (HCs) and individuals with other neurodegenerative disorders. Among the various miRNAs potentially involved, most of them showed comparable expression levels between ALS individuals and HCs, while others were found to be differentially expressed. Specifically, miR-15a-3p, miR-15a-5p, miR-150-5p, miR-182-5p, miR-192-3p, miR-221-3p, and miR-181a-2-3p were identified as dysregulated and have been previously implicated in neurodegenerative pathways [29–33]. In the present study we investigated the potential binding of those miRNAs to the HML-2 transcript, their expression in ALS individuals and controls and their modulatory effect on HML-2 expression.

## Methods

### Study Design

Combined case–control and in vitro study with retrospective data collection.

### Samples

The study was conducted in accordance with the principles outlined in the Declaration of Helsinki. All participants provided written informed consent prior to inclusion in the study. The study protocol was reviewed and approved by the local Ethics Committee at Sassari University Hospital (Azienda Ospedaliero-Universitaria, Sassari, Italy; IRB number 2395/2016), and all procedures involving human samples were performed in compliance with relevant institutional and regulatory guidelines.

Whole blood samples were collected from patients diagnosed with ALS and from healthy blood donors. A total of 13 ALS patients were recruited between March and October 2021 (5 females and 8 males; median age = 62 years) from ATSSardegna, UOS Malati ventilati e cure palliative ACA (Table 1). Samples from a group of 14 HCs were collected throughout 2021 at the Blood Transfusion Centre of Sassari (7 females and 7 males; median age = 61.5 years).

**Table 1:** Clinical characteristics of ALS patients

Samples	ALS 1	ALS 2	ALS 3	ALS 4	ALS 5	ALS 6	ALS 7	ALS 8	ALS 9	ALS 10	ALS 11	ALS 12	ALS 13
Sex	F	F	M	M	M	M	M	F	F	M	M	F	M
Age (y/o)	66	58	79	72	63	63	62	86	55	62	46	62	62
fALS/sALS	NR	s	NR	NR	s	s	NR	s	s	s	NR	NR	NR
Spinal/Bulbar	B	S	NR	NR	NR	NR	NR	B	NR	S	NR	NR	NR
Disease duration (mo)	24	108	96	72	12	24	36	120	60	192	NR	NR	NR
ALS-FRS	NR	< 20	< 20	< 20	NR	NR	NR	< 20	NR	< 20	NR	NR	NR
PEG/NIV	PEG	PEG/NIV	PEG	PEG	PEG	PEG	PEG	PEG	PEG	PEG	PEG	NR	NR
Tracheostomy	YES	YES	YES	YES	YES	YES	YES	YES	YES	YES	YES	NO	NR
Comorbidity	NR	NO	NO	NO	NO	Diabetes	NO	NO	Discoid Lupus Erythematosus	Diabetes	NO	NR	NR

**fALS** = familial amyotrophic lateral sclerosis; **sALS** = sporadic amyotrophic lateral sclerosis **F** = female; **M** = male; **NR** = not reported; **ALS-FRS** = functional rating scale; **PEG** = percutaneous endoscopic gastrostomy; **NIV** = non-invasive ventilation; **B** = bulbar onset; **L** = limb, **S** = spinal onset

### Blood Samples Collection

Peripheral venous blood samples were obtained from the participants using K2-EDTA tubes. The collected whole blood was gently layered over an equal volume of Ficoll (Sigma-Aldrich, St. Louis, MO, USA) in a 15 mL tube and centrifuged for 20 min at 1800 revolutions per minute (rpm) without brake. The peripheral blood mononuclear cells (PBMCs) were collected and stored at −80 °C in fetal bovine serum with 10% dimethyl sulfoxide for total RNA, including small RNAs, extraction.

### Identification of miRNAs able to Bind HERV-K Consensus Sequence

**Fig. 1:**
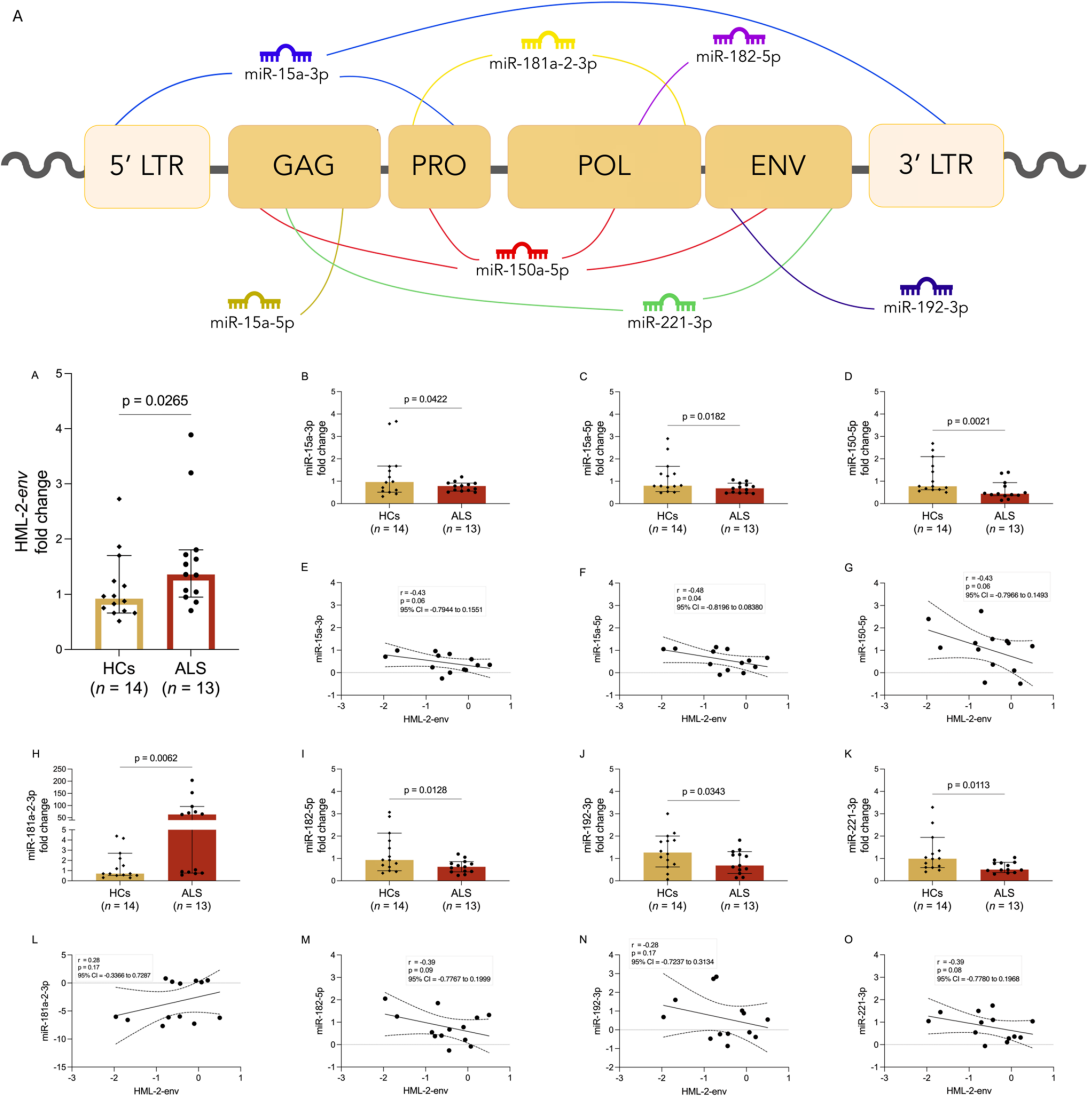
Representation of predicted miRNA binding sites on HML-2 and expression levels in ALS and HCs. Schematic representation of the predicted binding sites between miRNAs and the HML-2 transcript (**A**) HERV-K-env gene expression levels are upregulated in ALS individuals compared to controls (**B**). miR-15a-3p, miR-15a-5p, miR-150-5p, miR-182-5p, miR-192-3p, and miR-221-3p are downregulated in ALS patients (**C**, **D**,** E**, **J**, **K**, and **L**) and negatively correlated with the HERV-K envelope gene expression levels (**F**, **G**, **H**, **N**, **O** and **P**). miR-181a-2-3p was found to be upregulated in ALS patients (**I**) and positively correlated with HERV-K-env gene expression (**M**). Group comparisons were performed using the non-parametric Mann–Whitney U test. The ALS patient group comprised 13 individuals and the healthy control group 14 individuals (*n* = 13 ALS vs. *n* = 14 HC). No correction for multiple comparisons was applied, consistent with the targeted candidate-miRNA approach adopted in this study. Effect sizes are reported as Cohen's d with 95% confidence intervals (95% CI). Statistical significance was set at *p* < 0.05 (*)

To identify miRNAs with predicted binding affinity for the HML-2 transcript, a HML-2 consensus sequence [34] was submitted to the miRDB target prediction platform (https://mirdb.org). The resulting list was then filtered to only those miRNAs previously associated with neurodegenerative disorders, with particular focus on those implicated in ALS, based on literature. This filtering step yielded a panel of 16 candidate miRNAs. Expression profiles of these candidates were subsequently analyzed in total RNA extracted from PBMCs of ALS individuals and HCs, and seven of them showed differential expression between the two groups.

(Fig. 1). Two miRNAs, selected from the subset of downregulated candidates, were subjected to functional validation through co-transfection assays with a plasmid encoding the HML-2 consensus sequence, to evaluate their capacity to mediate post-transcriptional regulation.

### RNA Isolation, RT-PCR, and qPCR Analysis

Total RNA, including small RNAs, from PBMCs was purified using the miRNeasy Mini Kit (QIAGEN, Hilden, Germany) and treated with DNase to remove DNA contamination with the Turbo DNA-free kit (Thermo Fisher Scientific, Waltham, MA, USA), following manufacturer’s instructions. Then, RNA concentration was measured with a Nanodrop (Thermo Fisher Scientific, Waltham, MA, USA) and all the samples were adjusted to the same concentration. First-strand cDNA synthesis was performed using QuantiTect Reverse Transcription Kit (QIAGEN, Hilden, Germany). MiRNAs were reverse transcribed using miRCURY LNA RT Kit (QIAGEN, Hilden, Germany). No-RT (no Reverse Transcriptase) for each sample and no-TC (no Template Control) controls were made to check for DNA and reagent contamination, respectively. Subsequently, samples were analyzed in technical triplicates by real-time PCR using the QuantiNova SYBR Green PCR Kit (QIAGEN, Hilden, Germany) for mRNA quantification, and the miRCURY LNA SYBR Green PCR Kit (QIAGEN, Hilden, Germany) for miRNA analysis, according to manufacturer's instructions. The relative miRNA and mRNA expression levels were calculated by the 2^−∆∆Ct^ method. Normalization of mRNA expression levels was performed using HPRT1 as the reference gene. miRNA expression levels were normalized using the average of two reference small RNAs, snRNA-U6 and miR-103a-3p. Gene-specific primer pairs are listed in Table 2.

**Table 2:** Selected miRNAs capable of binding to HML-2 mRNA

Target Genes				
Accession Number	**Name**	**Target**	**References**	**Binding site on HML-2 sequence (nt)**
*MIMAT0004488*	hsa-miR-15a-3p	CAGGCCAUAUUGUGCUGCCUCA	[35–37]	369 (LTR), 3574 (PRO), 8306 (ENV), 8873 (LTR)
*MIMAT0000068*	hsa-miR-15a-5p	UAGCAGCACAUAAUGGUUUGUG	[36, 38–40]	369 (LTR), 3574 (PRO), 8306 (ENV), 8873 (LTR)
*MIMAT0000451*	hsa-miR-150-5p	UCUCCCAACCCUUGUACCAGUG	[41, 42]	1948 (GAG), 5824 (POL)
*MIMAT0004558*	has-miR-181a-2-3p	ACCACUGACCGUUGACUGUACC	[43, 44]	3558 (PRO), 3989 (POL), 4346 (POL), 6053 (POL)
*MIMAT0000259*	hsa-miR-182-5p	UUUGGCAAUGGUAGAACUCACACU	[45, 46]	4537 (POL), 5918 (POL), 6876 (ENV)
*MIMAT0004543*	hsa-miR-192-3p	CUGCCAAUUCCAUAGGUCACAG	[47]	7706 (ENV), 7958 (ENV), 8013 (ENV)
*MIMAT0000278*	hsa-miR-221-3p	AGCUACAUUGUCUGCUGGGUUUC	[48]	1512 (GAG), 7905 (ENV)
*MIMAT0004489*	hsa-miR-16–1-3p	CCAGUAUUAACUGUGCUGCUGA	[36, 49]	610 (LTR), 1743 (GAG), 1938 (GAG), 4941 (POL), 9114 (LTR)
*MIMAT0000077*	hsa-miR-22-3p	AAGCUGCCAGUUGAAGAACUGU	[50]	2235 (GAG), 6288 (ENV), 6550 (ENV), 8231 (LTR)
*MIMAT0004495*	hsa-miR-22-5p	AGUUCUUCAGUGGCAAGCUUUA	[50]	2235 (GAG), 6288 (ENV), 6550 (ENV), 8231 (LTR)
*MIMAT0004613*	hsa-miR-188-3p	CUCCCACAUGCAGGGUUUGCA	[37]	377 (LTR), 5159 (POL), 8881 (LTR)
*MIMAT0000457*	hsa-miR-188-5p	CAUCCCUUGCAUGGUGGAGGG	[37]	377 (LTR), 5159 (POL), 8881 (LTR)
*MIMAT0004683*	hsa-miR-362-3p	AACACACCUAUUCAAGGAUUCA	[37]	942 (LTR), 3478 (PRO), 9446 (LTR)
*MIMAT0002873*	hsa-miR-502-5p	AUCCUUGCUAUCUGGGUGCUA	[37, 51]	1265 (GAG), 1467(GAG), 3496 (PRO), 5096 (POL), 7134 (ENV)
*MIMAT0004797*	hsa-miR-582-3p	UAACUGGUUGAACAACUGAACC	[27, 37]	2270 (GAG), 3058 (GAG), 4421 (POL), 8091 (ENV)
*MIMAT0003247*	hsa-miR-582-5p	UUACAGUUGUUCAACCAGUUACU	[27, 37]	2270 (GAG), 3058 (GAG), 4421 (POL), 8091 (ENV)
*XM_047445854*	HML-2-env-FW HML-2-env-RV	CTGAGGCAATTGCAGGAGTT GCTGTCTCTTCGGAGCTGTT		
Reference Genes				
Accession Number	**Name**	**Target**		
*X59362*	U6	N/A – QIAGEN YP02119464		
*MIMAT0000101*	has-miR-103a-3p	AGCAGCAUUGUACAGGGCUAUGA		
*NM_000194*	HPRT1-FW HPRT1-RV	GCTATAAATTCTTTGCTGACCTGCTG AATTACTTTTATGTCCCCTGTTGACTGG		

The table reports the sequences of the selected miRNAs analyzed in this study, along with the primer sequences used for HML-2 detection and the nucleotide position at which each miRNA is predicted to bind the HML-2 consensus sequence. Reference genes employed for the normalization of miRNA and HML-2 transcript quantification are listed in the lower section

### Cell Culture

HEK293 cells were grown in Dulbecco’s Modified Eagle’s medium (DMEM) + GlutaMAX. [+] 4.5 g/L D-Glucose. [+] 110 mg/L Sodyum Pyruvate supplemented with 10% fetal bovine serum with 5% antibiotics and maintained in a tissue culture incubator at 37 °C with 5% CO_2_.

### Transient Co-Transfection of HEK-293

HEK-293 cells were grown in DMEM and maintained in a tissue culture incubator at 37 °C with 5% CO_2_. After 24 h of passaging and achieving approximately 80% confluence, HEK-293 cells were co-transfected with a HML-2 plasmid that has consensus sequence of the virus [8] and microRNAs (Thermo Fisher Scientific, Waltham, MA, USA) using the Lipofectamine 3000 kit (Thermo Fisher Scientific, Waltham, MA, USA).

Briefly, 1.5 µg of HML-2 plasmid or 1.5 µg of pcDNA (negative control), and 15 nM of miRNAs were mixed with Lipofectamine 3000, P3000, and Opti MEM reduced serum medium following manufacturer’s instructions (Thermo Fisher Scientific, Waltham, MA, USA). After 15 min of incubation at room temperature, the respective mixture was added to the cells. After 48 h of incubation, the cells were lysed, and the proteins and RNA were extracted.

### Protein Purification and Quantification

For protein extraction cells were lysed in NeuN buffer (Thermo Fisher Scientific, Waltham, MA, USA) supplemented with a protease inhibitor cocktail (MilliporeSigma, Burlington, MA, USA) and incubated for 10 min on ice. Subsequently, lysates were centrifuged at 10,000 g for 10 min at 4 °C. The supernatant, containing proteins, was transferred to new tubes and proteins were quantified using Pierce BCA Protein Assay Kit (Thermo Fisher Scientific, Waltham, MA, USA) according to manufacturer’s instructions.

### Western Blot Analysis

Protein concentration in the lysates was measured with the Pierce BCA protein assay kit and all samples to be compared were adjusted to the same concentration. 20 µg of protein per sample was mixed with lithium dodecyl sulfate buffer with 10% β-mercaptoethanol. Samples were denatured at 95 °C for 5 min at 900 rpm in a thermo shaker. They were then resolved in a 4–12% bis–tris gel (Invitrogen, Carlsbad, CA, USA) at 180 V for 45 min and transferred to a Polyvinylidene Difluoride (PDVF) membrane (iBlot PDVF stack; Thermo Fisher Scientific, Waltham, MA, USA) using an iBlot blotting system (Thermo Fisher Scientific, Waltham, MA, USA) for 7 min. Then, membranes were blocked with phosphate buffered saline (PBS) with 5% skimmed milk for 1 h and incubated at 4 °C with anti-HML-2 env primary antibodies (Austral Biologicals, San Ramon, CA, USA) (1:1000 in tris buffered saline (TBS) with 5% albumin and 0.02% sodium azide) overnight. The next day blots were washed 3 times in PBS-0.05% Tween-20 for 5 min and incubated with secondary anti-mouse IgG HPR-conjugated antibody (Cell Signaling Technology, Danvers, MA, USA) for 1 h (1:2500 in PBS-0.05% Tween-1% skimmed milk) in PBS. Blots were washed again 3 times and chemiluminescence was developed with Supersignal West Femto signal kit (Thermo Fisher Scientific, Waltham, MA, USA). Images were processed for quantification of chemiluminescence with Image J software.

### Statistical Analysis

Data was analyzed using GraphPad Prism version 8 (GraphPad Software, San Diego, CA, USA). The Kolmogorov–Smirnov test was applied to all data sets to assess normality. For normally distributed data, differences between groups were evaluated by one-way ANOVA followed by Dunnett’s post hoc test. For non-normally distributed data, comparisons were performed using the non-parametric Mann–Whitney U test, with Bonferroni correction for multiple comparisons. Each miRNA was analyzed as an individual, biologically selected candidate compared between patients and controls using the Mann–Whitney U test; given the targeted candidate-miRNA approach, rather than a large-scale exploratory screening, a global multiple-testing correction was not applied. Values in graphs are presented as mean ± standard error of the mean (SEM) for normally distributed data, and as median with interquartile range (IQR) for non-parametric data. Correlations between two variables were assessed using Spearman's rank correlation coefficient. Statistical significance was defined as *p* < 0.05.

## Results

The consensus sequence of HML-2 was analyzed using miRDB, yielding 288 microRNAs predicted to interact with the transcript. From this list, 16 candidates were selected based on previously reported associations with ALS or other neurodegenerative disorders (Table 2).

We then quantitatively evaluated the expression of these 16 miRNAs in RNA samples extracted from peripheral blood mononuclear cells of ALS individuals and HCs. Expression profiling, performed via qPCR, revealed a subset of miRNAs that were consistently downregulated in comparison with HC samples. Two miRNAs, selected among the most significantly downregulated, were chosen for functional validation. Each of the two candidate miRNAs was co‑transfected into HEK‑293 cells alongside a construct containing the HML‑2 consensus sequence.

We investigated the expression levels of HML-2-*env* and miRNAs in PBMC of ALS patients and HCs by qPCR. The binding sites on the HML-2 sequence of the miRNAs that showed a differential expression in ALS patients compared to controls are depicted in Fig. 1A. There was upregulation of HML-2-*env* transcript in patients compared to controls (*p* = 0.02) (Fig. 1B). While levels of most miRNAs were found to be similarly expressed in ALS patients and HCs (supplementary materials, Figure S1) a subset showed significant downregulation in ALS, including miR-15a-3p (*p* = 0.04; Fig. 1C), miR-15a-5p (*p* = 0.01; Fig. 1D), miR-150-5p (*p* = 0.001; Fig. 1E), miR-182-5p (*p* = 0.012; Fig. 1J), miR-192-3p (*p* = 0.034; Fig. 1K), and miR-221-3p (*p* = 0.011; Fig. 1L). In contrast, miR-181a-2-3p was significantly upregulated in ALS compared to controls (*p* = 0.006; Fig. 1I). To assess whether the observed miRNA alterations reflect a coordinated dysregulation pattern, a composite miRNA score was calculated for each subject as the mean expression of the downregulated candidate miRNAs (miR-15a-3p, miR-15a-5p, miR-150-5p, miR-192-3p, and miR-221-3p). Comparison between ALS patients and HCs using the Mann–Whitney test revealed a significant difference (*p* = 0.019), supporting the presence of a coordinated dysregulation of these miRNAs in ALS (supplementary materials, figure S2).

Moreover, the expression levels of some of these miRNAs showed negative correlations with HML-2-*env*, suggesting a potential inverse relationship between reduced miRNA levels and increased HML-2 expression. Specifically, among the analyzed miRNAs, HML-2-*env* expression levels showed a statistically significant negative correlation exclusively with miR-15a-5p (*r* = −0.48, *p* = 0.04; Fig. 1G). Although not statistically significant, we found trends toward negative correlation with miR-15a-3p (*r* = −0.43, *p* = 0.06; Fig. 1F), miR-150-5p (*r* = −0.43, *p* = 0.06; Fig. 1H), miR-182-5p (*r* = −0.39, *p* = 0.09; Fig. 1N), miR-192-3p (*r* = −0.28, *p* = 0.17; Fig. 1O), and miR-221-3p (*r* = −0.39, *p* = 0.08; Fig. 1P). In contrast, a tendency toward a positive correlation was observed only between miR-181a-2-3p and HML-2-*env* (*r* = 0.28, *p* = 0.17; Fig. 1M).

**Fig. 2:**
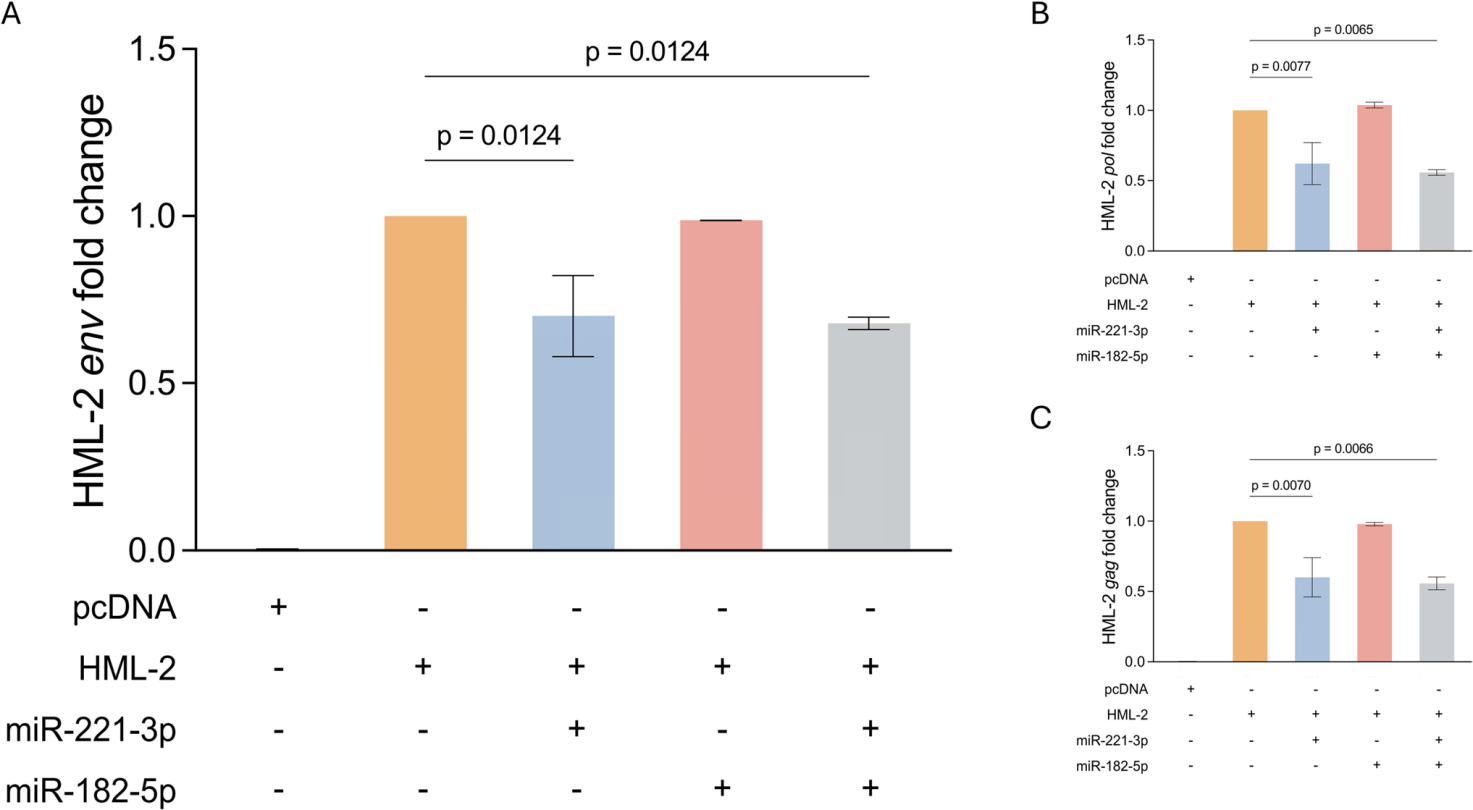
miR-182-5p and miR-221-3p synergistically modulate env gene expression in HEK-293 cells following transfection. HEK-293 cells were transfected with pcDNA, HML-2 alone or co-transfected with HML-2 + miR-182-5p, miR-221-3p or with both miRNAs. Total RNA was extracted post-transfection and analyzed by qPCR to assess the HML-2-*env* (**A**), *pol* (**B**) and *gag* (**C**) gene expression. Statistical significance was set at *p* < 0.05 (*)

**Fig. 3:**
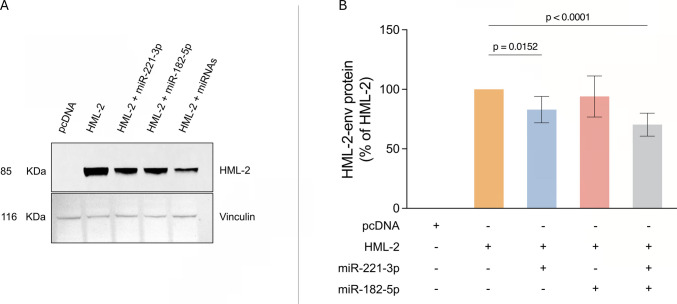
miR-182-5p and miR-221-3p synergistically modulate HML-2 envelope protein expression in HEK-293 after transfection. Proteins purified from HEK-293 cell line transfected with pcDNA, HML-2 alone or co-transfected with HML-2 + miR-182-5p, miR-221-3p or with both miRNAs were analyzed by western blot. Protein expression levels were assessed by western blot analysis and protein signals were quantified using ImageJ and plotted (**B**). (**A**) Representative image of a blot. (**B**) Comparison of HERV-K-env protein levels among the different treatments. Data are derived from three independent biological replicates. Statistical significance was set at *p* < 0.05 (*)

To investigate the potential role of miRNAs in regulating HML-2 expression we co-transfected cells with an HML-2 plasmid and miR-182-5p and miR-221-3p and measured HML-2 transcripts two days after transfection (Fig. 2). These two miRNAs are dysregulated in various neurological diseases, including ALS [29, 30, 45, 52–55], and have been implicated in the regulation of multiple cellular processes such as apoptosis, cell proliferation, and differentiation [56, 57]. The levels of HML-2 RNA were lower in the cells co-transfected with miR-221-3p and with the combination of both miRNAs but not with miR-182-5p alone (Fig. 2A). The same effect was observed on HML-2*-gag* and *-pol* transcripts (Fig. 2B and C), suggesting that the miRNA may regulates HML-2 at the pre-mRNA stage. Given the documented neurotoxic role of the envelope protein in ALS, associated with neuroinflammatory responses and motor neuron damage HEK293 cells were selected due to their high transfection efficiency and well-characterized molecular background, which allows controlled assessment of exogenous miRNA-target interactions with reduced experimental variability, we performed env-protein-level validation. Co-transfection with the individual miR-221-3p decreased the level of HML-2 env protein and when the miRNAs were used in combination, we found a synergistic effect (Fig. 3). No significant effect on the protein was found for miR-182-5p alone. The concordance between the RNA and the protein results indicate that miR-221-3p downregulates the target gene, most likely by inhibiting its translation or reducing its stability. (Fig. 3) (supplementary materials, figure S3 for the uncropped membrane images). One possibility to explain the synergistic effect is that miR-182-5p binding might cause a change on the secondary structure of the HERV-K mRNA making it more accessible to miR-221-3p binding and enhancing its ability to suppress translation or reduce mRNA stability (Fig. 3A and B). However, this remains an open question and requires experimental validation. These results suggest that the use of specific miRNAs or their combination could represent a potential therapeutic strategy for the regulation of HML-2 expression.

## Discussion

HML-2 reactivation has been observed in several pathological conditions, but the molecular factors responsible for this process are still not fully understood. In this study, we explored whether specific miRNAs are involved in modulating HML-2 expression.

MiRNAs play essential roles across various biological contexts, far beyond the classical model of post-transcriptional gene silencing through binding to the 3′ untranslated region (3′ UTR) of target mRNAs, resulting in gene silencing [58, 59]. Binding sites of miRNAs have also been identified in other regions of mRNA, including the 5′ UTR and coding sequences, resulting in gene silencing [60], and binding to the promoter regions, resulting in transcription induction [61, 62]. In addition to regulating host gene expression, miRNAs play a significant role in modulating genes of infecting viruses or bacteria. Notably, they have been implicated in the control of HIV-1 latency and reactivation, either contributing to the maintenance of transcriptional silencing of the integrated provirus or promoting its reactivation, depending on the cellular context and the specific miRNA expression profile [63–65]. In light of the multiple roles attributed to microRNAs, we propose that miRNA-mediated regulation could represent a potential mechanism contributing to HML-2 reactivation. In fact, they can be involved in neuronal development, synaptic plasticity, and neural circuit maintenance [66]. Several miRNAs are enriched in the central and peripheral nervous systems, where they modulate the expression of genes involved in neurogenesis, axonal growth, and neuromuscular signaling [67–69]. Notably, specific miRNAs have been associated with motor neuron integrity and have emerged as potential contributors to the pathophysiology of neurodegenerative disorders such as ALS [70, 71].

Members of the miR-15/16 cluster, located within the intronic region of the DLEU2 gene, have shown variable expression profiles in ALS, with both up- and downregulation described in blood, muscle, and neuron-derived extracellular vesicles [36, 72, 73]. These miRNAs target Bcl-2, an anti-apoptotic protein involved in neuronal survival, suggesting a mechanistic link to ALS-related neurodegeneration [74–76]. MiR-150-5p has been found significantly downregulated in plasma from sporadic ALS patients [41], and miR-181 has been proposed as a prognostic biomarker, with higher plasma levels predicting reduced survival, in combination with neurofilament light chain [43]. In contrast, although no specific molecular mechanisms have been proposed so far for miR-182 and miR-192 in the context of ALS, both miRNAs have been found to be deregulated in the disease [37], as confirmed by our findings. MiR-182, part of the neuron-enriched miR-183/96/182 cluster, has been shown to regulate axonal growth and dendritic maturation via the AKT/PTEN pathway, and to interact with TDP-43 and FUS in other neurodegenerative contexts [45, 46]. MiR-192-5p has been investigated in models of cerebral ischemia, where it shows neuroprotective and anti-inflammatory effects by downregulating Dyrk1a expression [77]. Given the shared features of neuroinflammation and neuronal loss, similar mechanisms may also be relevant to the pathophysiology of ALS. To further support the robustness of our findings, we interrogated an independent publicly available dataset (GEO: GSE148097). Most investigated miRNAs showed a consistent directional trend with our cohort, with the exception of hsa-miR-181a-2-3p, whose upregulation in our data is consistent with its reported role as a prognostic biomarker in ALS [43]. These observations support the biological relevance of the identified miRNA signature, although larger cohorts and standardized pipelines will be required to fully validate its diagnostic and prognostic value in ALS.

Beyond their known functions, it is particularly noteworthy that our findings revealed the ability of these miRNAs to bind the HML-2 transcript, suggesting a potential regulatory interaction that may contribute to the control of HERV-K expression.

Growing evidence points to the HML-2 potential contribution to disease pathogenesis. High expression levels of HML-2-env have been identified in brain samples obtained post-mortem from subsets of patients [8, 78]. The envelope protein has been identified within extracellular vesicles derived from the plasma of ALS patients, with increased levels observed in the advanced stages of the disease [79]. Recently, it was demonstrated that individuals with ALS exhibit higher levels of HML-2-env protein in cerebrospinal fluid [9] and corresponding antibodies in plasma than control subjects. Intriguingly, a diminished humoral response against HML-2 was observed in patients at later stages of the disease. This decrease in antibody levels was also correlated with reduced predicted survival rates suggesting that the reactivation of HML-2 may play a role in the pathogenesis of ALS and that a humoral response against it could be protective for patients, whereas a loss of this response might be associated with a poorer prognosis [80]. Furthermore, antibody levels against HML-2-env appear to correlate with those detected against TDP-43 [81], whose aberrant protein aggregation and localization play a critical role in ALS [82]. Notably, we documented that HML-2 possesses the capacity to modulate the immune system, primarily by inducing mediators involved in the pro-inflammatory response [83].

The mechanisms underlying the regulation of HML-2 expression remain largely unknown. However, given its consistent upregulation in various pathological contexts one potential approach to understanding its role is to investigate how its expression is controlled. Since miRNAs predominantly regulate the expression of target genes negatively, we hypothesize that miRNAs’ dysregulation may lead to a failure in the silencing of HML-2 and its consequent overexpression.

In our analysis, we identified a consistent downregulation of miR-15a-3p, miR-15a-5p, miR-150-5p, miR-182-5p, miR-192-3p, and miR-221-3p in ALS patients relative to healthy controls, alongside a significant upregulation of miR-181a-2-3p. These findings suggest that specific miRNA dysregulation may actively contribute to disease pathogenesis by modulating key cellular processes, including apoptosis, neuroinflammation, and neuronal homeostasis [29, 43, 84–86]. Notably, while each of these miRNAs has been individually linked to distinct molecular pathways, their coordinated deregulation in ALS implies a broader disruption of post-transcriptional gene regulation rather than isolated molecular events. Among the downregulated miRNAs, miR-182-5p has been implicated in neuronal structural integrity and axonal growth [29], while miR-221-3p has been described as neuroprotective under cellular stress conditions, with evidence supporting its role in suppressing neuronal apoptosis and attenuating pro-inflammatory signaling [87]. Although the function of miR-192-3p remains incompletely characterized, emerging data suggest its involvement in the modulation of pro-inflammatory cytokine signaling [86], warranting further investigation of its potential role in the neuroinflammatory setting of ALS. Conversely, the upregulation of miR-181a-2-3p observed in our cohort is consistent with previous reports of elevated circulating miR-181 levels in ALS, where higher expression has been associated with accelerated disease progression and shorter survival [85]. Whether this upregulation represents a primary pathological event or a secondary compensatory response remains to be determined. Although weak, probably due to the limited number of samples, we found a negative correlation between the expression levels of miRNAs and those of HML-2-*env*, which is upregulated in patients compared to controls. In the correlation analysis, we observed that, unlike the others, miR-181a-2-3p shows a trend towards a positive correlation with HML-2 gene expression levels. The weak positive correlation suggests that this miRNA, although capable of binding the HML-2 transcript in silico, is unlikely to participate in the downregulation of HML-2, as both its expression and HML-2 *env* levels are increased in ALS patients. Nevertheless, the possibility that it may contribute to retroviral reactivation, rather than silencing, cannot be excluded. In addition, we documented that miR-221-3p can reduce HML-2 expression possibly by inhibiting translation or decreasing mRNA stability, as evidenced by the reduced levels HML-2 transcripts, especially the envelope gene, and the envelope protein. Conversely, reduced levels of miR-221-3p may result in augmentation of expression of HERV-K by relieving this post-transcriptional regulation. All the investigated miRNAs are involved in neurodegenerative processes, but for the first time, we suggest a potential role in the regulation of HML-2. It remains unclear why miRNAs are downregulated in ALS. One plausible mechanism involves the loss of TDP‑43 function, a key regulator of miRNA biogenesis. Under physiological conditions, TDP‑43 interacts with the Drosha and Dicer complexes to facilitate miRNA processing [88, 89] both at the nuclear and cytoplasmic levels. However, in ALS, TDP‑43 frequently accumulates in the cytoplasm and forms aggregates, preventing it from carrying out its normal nuclear functions and thereby interfering with the miRNA biogenesis machinery. In parallel, increased HML‑2 expression has been shown to repress *ASRGL1* [90], a gene involved in proteostasis maintenance. *ASRGL1* downregulation leads to the accumulation of aberrantly modified proteins, including TDP‑43, further promoting its pathological aggregation. This creates a potential self-reinforcing loop in which miRNA downregulation permits HML-2 activation, which in turn suppresses *ASRGL1*, leading to increased TDP-43 pathology and further impairment of miRNA biogenesis. Such a feedback mechanism may contribute to the selective downregulation of specific miRNAs observed in ALS and could underlie the dysregulated retroelement expression seen in this disease. However, this proposed cascade remains hypothetical and requires experimental validation.

## Conclusions

These findings raise several hypotheses that require further validation. Confirming the existence of a feedback loop involving TDP-43 dysfunction, altered miRNA processing, and HML-2 activation will be important to better understand its relevance in ALS pathology. Despite the relevance of these findings, several limitations should be acknowledged in the interpretation of the present study. Importantly, our findings from PBMCs should be interpreted as peripheral correlates of disease-associated molecular alterations, rather than direct evidence of CNS mechanisms. The exact mechanisms through which these miRNAs might inhibit the HML-2 expression are not yet defined. The correlation observed between HML-2 env expression and miRNAs suggests a possible link, but this needs to be confirmed through further experiments. Additionally, we do not know which miRNA has the greatest influence on HML-2 expression. The selected miRNAs might act individually or, more likely, have a synergistic effect on the regulation of HML-2. However, fully understanding HML-2 regulation strategies could pave the way for new therapeutic strategies to improve patients' conditions.

## Supplementary Information

Below is the link to the electronic supplementary material.

ESM 1 (DOCX 586 KB)

## Data Availability

Data sharing is not applicable to this article as no datasets were generated or analysed during the current study.

## Abbreviations

ALS: Amyotrophic lateral sclerosis
HERV: Human Endogenous Retrovirus
Env: Envelope
miRNAs: MicroRNAs
ncRNAs: Non-coding RNAs
fALS: Familial ALS
sALS: Sporadic ALS
HCs: Healthy controls
ALS-FRS: Functional rating scale
PEG: Percutaneous endoscopic gastrostomy
NIV: Non-invasive ventilation
PBMCs: Peripheral blood mononuclear cells
No-RT: No Reverse Transcriptase
No-TC: No Template Control
DMEM: Dulbecco’s Modified Eagle’s medium
PDVF: Polyvinylidene Difluoride
PBS: Phosphate buffered saline
TBS: Tris buffered saline
SEM: Standard error of the mean
IQR: Interquartile range
3′ UTR: 3′ Untranslated region
5′ UTR: 5′ Untranslated region
