# Supplementary material for: Downregulation of miRNAs Accompanies Increased HERV-K (HML-2) Expression in Amyotrophic Lateral Sclerosis
Source: Mol Neurobiol. 2026 Apr 17;63(1):570. doi: 10.1007/s12035-026-05861-5 (PMC13090184; doi:10.1007/s12035-026-05861-5)
Supplement: Supplementary file 1 — (DOCX 586 KB) [file 12035_2026_5861_MOESM1_ESM.docx]

HERV-K, subtype HML-2, expression inversely correlates with and is regulated by miRNAs expression in amyotrophic lateral sclerosis

Elena Rita Simula, Ph.D.,^1*^ Marta Garcia-Montojo, Ph.D.,^2,3*^ Mattia Canu, MS,^4^ Vanna Chessa, MD,^4^ Tommaso Ercoli, MD,^5^ Elisa Ruiu, MD,^5^ Paolo Solla, MD^5^, Avindra Nath, MD,^2†^ Leonardo Antonio Sechi, Ph.D.^1,6†^

^1^ Department of Biomedical Sciences, Division of Microbiology and Virology, University of Sassari, Sassari, Italy.

^2^ National Institute of Neurological Disorders and Stroke, National Institutes of Health, Bethesda, MD, USA.

^3^ Twilight Bioscience, Inc. 100 Cummings Center. Suite 207-209. Beverly, MA. 01915, USA.

^4^ ASL Sassari, SC Anestesia Territoriale Cure Palliatiave, 07100 Sassari, Italy.

^5^ Neurological Unit, AOU Sassari, University of Sassari, Viale S. Pietro 10, Sassari, 07100, Italy.

^6^ Complex Structure of Microbiology and Virology, University Hospital of Sassari, Sassari, Italy.

^*^ These authors contributed equally

^†^ Corresponding author: [sechila@uniss.it](mailto:sechila@uniss.it); [avindra.nath@nih.gov](mailto:avindra.nath@nih.gov).

**Supplementary material**

**Figure S1. Expression levels of HML-2-env and miRNAs in ALS patients and HCs.**

Expression levels of miR-16-1-3p, miR-22-3/5p, miR-188-3/5p, miR-362-3p, miR-502-5p, and miR-582-3/5p in ALS patients compared to healthy control (Fig. **A**, **B**, **C**, **D**, **E, F, G, H, I**). Statistical significance was set at p < 0.05 (*).

**Figure S2. Composite miRNA score in ALS patients and healthy controls (HCs).**

Composite miRNA score in ALS patients and healthy controls (HCs). The score was calculated as the mean expression of five downregulated candidate miRNAs (miR-15a-3p, miR-15a-5p, miR-150-5p, miR-192-3p, and miR-221-3p) for each subject. Violin plots display the distribution of the composite score in ALS patients (red) and HCs (yellow); horizontal lines indicate the median and interquartile range (IQR). Comparison between groups was performed using the Mann-Whitney U test. A statistically significant difference was observed between ALS patients and HCs (median 0.70 vs. 0.95; Hodges-Lehmann estimate = -0.36, 95% CI: -1.05 to -0.06; p = 0.019), supporting a coordinated dysregulation of the selected miRNAs in ALS. Individual data points are shown. Statistical significance was set at p < 0.05 (*).

**Figure S3. Full uncropped Western blot images.**

Full, uncropped images of the Western blot membranes corresponding to the three independent biological replicates are provided. In each blot, the upper band corresponds to the full-length HML-2 Env protein (85 KDa) and represents the signal considered for the analyses reported in the manuscript. The lower band (45 KDa) corresponds to the transmembrane (TM) subunit and was not included in the quantitative analyses.
